# Discovery of Leptulipin, a New Anticancer Protein from theIranian Scorpion, *Hemiscorpius lepturus*

**DOI:** 10.3390/molecules27072056

**Published:** 2022-03-22

**Authors:** Ali Rezaei, Saeme Asgari, Samira Komijani, Seyedeh Narjes Sadat, Jean-Marc Sabatier, Davood Nasrabadi, Kamran Pooshang Bagheri, Delavar Shahbazzadeh, Mohammad Reza Akbari Eidgahi, Michel De Waard, Hasan Mirzahoseini

**Affiliations:** 1 Department of Biotechnology, Faculty of Medicine, Semnan University of Medical Sciences, Semnan 3514799422, Iran; alirezaei3333@yahoo.com (A.R.); davood_bn@yahoo.com (D.N.); 2 Venom and Biotherapeutics Molecules Laboratory, Medical Biotechnology Department, Biotechnology Research Center, Pasteur Institute of Iran, Tehran 1316943551, Iran; s.komijani6267@gmail.com (S.K.); narges.sadat2009@gmail.com (S.N.S.); shahbazzadeh@yahoo.com (D.S.); 3 Department of Biochemistry and Biophysics, Faculty of Advanced Sciences and Technology, Tehran Medical Sciences, Islamic Azad University, Tehran 5157944533, Iran; asgari.saeme@yahoo.com; 4 Institute of NeuroPhysiopathology (INP), Faculté de Pharmacie, Université D’Aix-Marseille, UMR 7051, 27 Bd Jean Moulin, CEDEX 05, 13385 Marseille, France; sabatier.jm1@libertysurf.fr; 5 L’Institut du Thorax, INSERM, CNRS, University of Nantes, 44000 Nantes, France; 6 LabEx “Ion Channels, Science & Therapeutics”, 65560 Valbonne, France; 7 Smartox Biotechnology, 6 Rue Des Platanes, 38120 Saint-Egrève, France

**Keywords:** venom, anticancer protein, colorectal cancer, breast cancer

## Abstract

Cancer is one of the leading causes of mortality in the world. Unfortunately, the present anticancer chemotherapeutics display high cytotoxicity. Accordingly, the discovery of new anticancer agents with lower side effects is highly necessitated. This study aimed to discover an anticancer compound from *Hemiscorpius lepturus* scorpion venom. Bioactivity-guided chromatography was performed to isolate an active compound against colon and breast cancer cell lines. 2D electrophoresis and MALDI-TOF were performed to identify the molecule. A partial protein sequence was obtained by mass spectrometry, while the full-length was deciphered using a cDNA library of the venom gland by bioinformatics analyses and was designated as leptulipin. The gene was cloned in pET-26b, expressed, and purified. The anticancer effect and mechanism action of leptulipin were evaluated by MTT, apoptosis, and cell cycle assays, as well as by gene expression analysis of apoptosis-related genes. The treated cells displayed inhibition of cell proliferation, altered morphology, DNA fragmentation, and cell cycle arrest. Furthermore, the treated cells showed a decrease in *BCL-2* expression and an increase in *Bax* and *Caspase 9* genes. In this study, we discovered a new anticancer protein from *H. lepturus* scorpion venom. Leptulipin showed significant anticancer activity against breast and colon cancer cell lines.

## 1. Introduction

Throughout the world, cancer is among the top causes of death. Over the past two decades, cancer death rates have decreased significantly in developed countries, but they remain a highly important public health problem in many developing countries [1]. Cancer is characterized by prominent features including: increased cell proliferation, escape of growth inhibition, and high resistance to cell death [2]. Combining chemotherapy, radiation therapy, and surgery has proven to be the gold standard in cancer treatment, but it is accompanied by devastating side effects and toxicity, as well as a low degree of specificity [3]. Additionally, many classic anticancer drugs are unable to distinguish between normal and cancerous cells, which can lead to systemic toxicity and various side effects [4] although some efforts have accomplished the lowering of the toxicity of chemotherapeutics or have found a new way to comply with cancer such as ferroptosis [5,6,7]. It is imperative to discover new drugs with high selectivity and specificity to deal with this problem. Natural resources have the potential to help the discovery of new promising anticancer agents that lack side-effect toxicity [8]. Proteins or peptides may be interesting anticancer agents, as they act specifically against their targets to induce apoptosis [9,10,11].

According to the abovementioned problems, recent studies have been focusing on the discovery of natural anticancer compounds derived from the venom of venomous animals such as scorpions [10,12,13]. Traditional medicine has used venom as a therapeutic source against different diseases such as epilepsy, rheumatism, and cancer for a long time [14,15]. Scorpion venoms are composed of many peptides or proteins such as ctriporin, chlorotoxins (cltx), and neopladine I and II [16,17,18]. In related applications, several studies showed that scorpion venoms inhibit proliferation in vivo and in vitro, as well as inducing apoptosis [19,20,21,22]. Research studies have identified a protein of high molecular weight (72 kDa) named bengalin from the venom of the *Heterometrus bengalensis Koch* scorpion that induces apoptosis in human leukemic cells with the participation of pro- and anti-apoptotic proteins [23]. Another protein extracted from *Mesobuthus martensii Karsch*, now produced in a recombinant form, is the analgesic-antitumor peptide (rAGAP). which inhibits the proliferation and migration of SHG-44 cells, leading to an arrest in the G1 phase [24]. The peptides neopladine 1 and 2 have been identified from the venom of *Tityus discrepans* scorpion and induce apoptosis in SKBR3 cells by increasing expression of *FasL* and *BcL-2* [25].

Scorpions are among the oldest animals on earth. More than 1500 species of scorpions are grouped into 18 families phylogenetically. One of the deadliest scorpions in Iran is *Hemiscorpius lepturus* (*H. lepturus*), which belongs to the Scorpionidae family [26]. Its venom contains many peptides and proteins with some molecules possessing therapeutic potential such as hemicalcin, hemitoxin, leptucin, hemilipin, discovered earlier [27,28,29,30]. In the present study, we aimed to identify a new anticancer protein from this Iranian scorpion venom and evaluate its potential to induce apoptosis in breast and colorectal cancer cells.

## 2. Material and Methods

### 2.1. Reagents, Media, Bacteria and Cells

*H. lepturus* scorpion venom was gifted by Dr. D. Shahbazzadeh. Sodium dodecyl-sulfate polyacrylamide gel electrophoresis (SDS-PAGE) and Two-dimensional gel electrophoresis (2-DE) materials, Sephadex G 50, Hoechst 33,258, Penicillin–Streptomycin Solution, 3, 3′-Diaminobenzidine (DAB), and 3-(4,5-Dimethyl-2-thiazolyl)-2,5-diphenyl-2H-tetrazolium bromide (MTT)s were purchased from Sigma-Aldrich (Saint Louis, MO, USA). Acetonitrile and trifluoroacetic acid (TFA) were obtained from Merck (Kenilworth, NJ, USA). HEK293, HT-29, and MDA-MB-231 cells were purchased from the Pasteur Institute of Iran. DMEM, DMSO, and fetal bovine serum (FBS) were purchased from Gibco, Life Technologies (Grand Island, NY, USA).

### 2.2. Graphical Representation of the Study

**Scheme 1:**
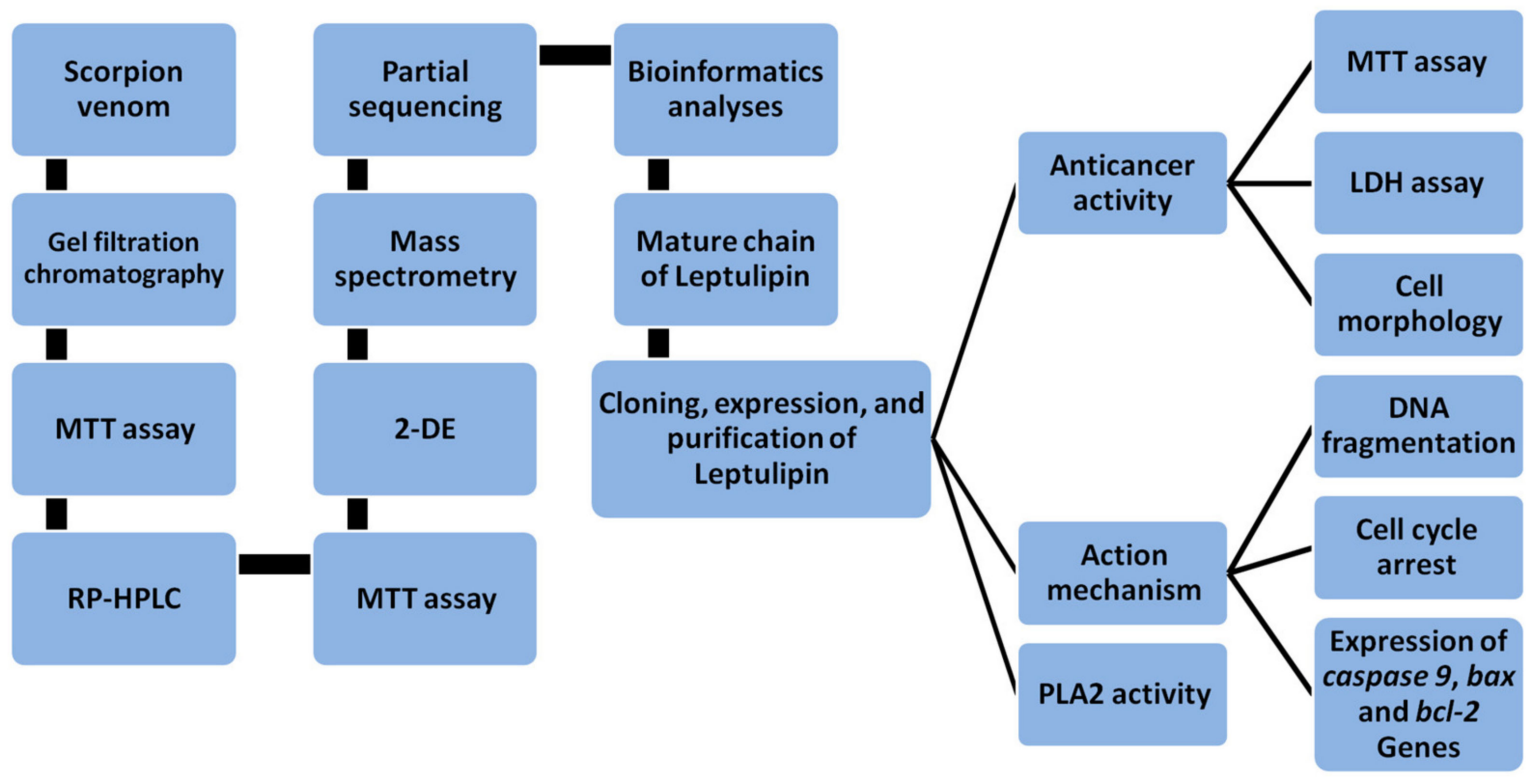
Graphical presentation of the methodology.

The methodology of this paper was abstracted as a diagram to demonstrate the workflow of experiments and analyses (Scheme 1).

### 2.3. Venom Preparation

The venom was dissolved in distilled water by a vortex mixer for 2 min, centrifuged at 10,000× *g* for 15 min, and the supernatant stored at −20 °C until use. The venom concentration was determined by Bradford method.

### 2.4. Venom Fractionation

At first, the venom was fractionated by gel filtration chromatography using an Akta purifier 10 (GE healthcare, Chicago, IL, USA) and a Sephadex G50 column (Sigma-Aldrich, Saint Louis, MO, USA). Equilibration and elution were performed using 20 mM ammonium acetate (pH 6.8) at a flow rate of 3 mL/min. The eluted fractions were lyophilized and kept frozen in −20 °C until examined for anticancer activity. Sodium dodecyl sulfate-polyacrylamide gel electrophoresis (SDS-PAGE) was performed to determine the molecular weight of the proteins in the fractions. The fractions were subjected to SDS-PAGE using 12% polyacrylamide at 20 mV for 2 h. Finally, the gel was stained with Coomassie Brilliant Blue R-250 (BioRad, Hercules, CA, USA).

In the next step, the fraction with the highest anticancer activity was purified by a reverse phase-high performance liquid chromatography (RP-HPLC) system (Knauer, Berlin, Germany) using a C18 column (250 mm × 4.6 mm, 5 mm; Beckman, Brea, CA, USA). Solvent A was 0.1% TFA in water and solvent B was acetonitrile containing 0.1% TFA. The elution was performed at the flow rate of 1.0 mL/min with an acetonitrile linear gradient ranging 20–70% over 45 min. The fractions were collected and used for anticancer activity assay. The eluted fractions were collected, lyophilized, and kept frozen at −20 °C until examined for anticancer activity. SDS-PAGE was performed as aforementioned above.

### 2.5. Evaluation of the Anticancer Activity of Venom Fractions

The HT-29 (colon cancer) and MDA-MB-231 (breast cancer) cells were grown in DMEM (Dulbecco’s Modified Eagle Medium), supplemented with 10% heat inactivated fetal bovine serum and 10 μL/mL penicillin–streptomycin solution. The cells were incubated at 37 °C, 5% CO_2_, and humidified atmosphere.

The cytotoxicity of gel filtration- and HPLC-derived fractions on cancer cells were assessed by MTT assay. Briefly, the cells were seeded in a 96-well plate at density of 3 × 10^4^ cells/well. Before exposure to the fractions, the culture medium was changed to FBS-free medium and incubated with different concentration of fractions for 24 h; then 10 μL of MTT solution (5 mg/mL) was added to each well and incubated at 37 °C for 4 h. The medium was removed, 100 μL of dimethylsulfoxide (DMSO) was added, and the cells were incubated in a dark place at room temperature for 20 min. Finally, the absorbance was measured at 570 nm by a microplate spectrophotometer (Epoch, Biotek Co., Bothell, WA, USA).

### 2.6. Two-Dimensional Gel Electrophoresis

2-DE was performed as detailed earlier [28]. Isoelectric focusing (IEF) carried out by using a 10-cm immobilized gradient (IPG) strips (pH 3–10, non-linear). The fractions were dissolved in a rehydration solution including 8 M urea, 2% 3-[(3-cholamidopropyl)-dimethylammonio]-1-propanesulfonate (CHAPS), 0.5% IPG buffer, 0.002% bromophenol blue and 1 M dithiothreitol (DTT) and then applied to the IPG strip. IEF was performed at 300 V using an IPGphor at 20 °C. In the second dimension, strips were equilibrated with DTT and iodoacetamide for 20 min and then diluted in 5 mL of equilibration buffer solution (6 M urea, 2% SDS, 30% glycerol, 50 mM Tris-HCl, pH 7.4, 0.002% bromophenol blue). Then, the strip was loaded to a 15% polyacrylamide gel and run at 100 V for 4 h. The gel was stained with Coomassie Blue G-250.

### 2.7. Mass Spectrometry

In order to identify proteins, the spots were cut out and sent to an external facility (York University, Mass Spectrometer facility, Heslington, UK). Then, an in-gel digestion method was performed with trypsin (Promega, Madison, WI, USA). The obtained peptide fragments of each spot were subjected to a Bruker Ultrafex III-MALDI-TOF/TOF mass spectrometer (Bruker Co., Billerica, MA, USA) for partial sequencing. The experimental spectrum was compared to the theoretical spectrum by using Mascot server (https://www.matrixscience.com/search_form_select.html accessed on 10 August 2021) from proteins available in the SwissProt database.

### 2.8. Bioinformatics Analyses

#### 2.8.1. Similarity Analysis and Determination of the Mature Chain

BLASTP search (https://blast.ncbi.nlm.nih.gov/Blast.cgi?PAGE=Proteins accessed on 11 August 2021) was performed to analyze the similarity of peptide fragments derived from spots against the NCBI and Uniprot databank, as well as the cDNA library of *H. lepturus* (accn: SRX1584448, https://www.ncbi.nlm.nih.gov/sra/SRX1584448 accessed on 12 August 2021). The best hits in terms of identity were selected for further analysis. The first hit (spot 1) was selected as our desired protein for gene cloning and expression.

To define the signal peptide, mature chain, and propeptide for spot 1, several sequences of which the gene organization had already been identified [31,32,33,34,35,36,37] were selected for multiple alignments by Clustal Omega server (https://www.ebi.ac.uk/Tools/msa/clustalo/ accessed on 13 August 2021).

To control the accuracy of alignment, the evolutionary signature of aligned peptides was determined as well. Then the molecular weight of the mature chain was calculated using the ‘Protparam’ server (http://web.expasy.org/protparam) (accessed on 14 August 2021).

#### 2.8.2. Prediction of 3D Structure of the Protein

The 3D structure of phospholipase A2 was predicted using the Iterative Threading Assembly Refinement (I-TASSER) server at Michigan University [38]. A model with the highest c-score and lowest root mean square deviation (RMSD) was selected. This model was viewed and analyzed by using ‘UCSF Chimera’ software package. This predicted structure was aligned against honey bee (*Apis melifera*) PLA2 structure (1POC) and visualized using the UCSF Chimera software package [39]. PLA2 from honey bee venom was selected, as it is the only PLA2 which its 3D structure is the most similar to other scorpion PLA2s [31,32,33].

### 2.9. Construction of Plasmid, Expression and Purification of Leptulipin

The DNA sequence corresponding to the mature chain of the candidate protein was codon-optimized for *E. coli* cell expression. A sequence containing an N-terminal his-tag flanked by *Nde*I and *Xho*I was synthesized by an external facility (Biomatik Co., Kitchener, ON, Canada), and cloned into pET-26b (+) vector (Invitrogen, California, United States). The vector was transformed into *E. coli BL21* (DE3) *Plys*S. Bacterial cultures were grown in a shaking incubator in LB broth medium at 37 °C overnight. IPTG (1 mM) was added to the suspension when the cell density reached 0.6 at 600 nm. The cells were incubated at 28 °C for 8 h. The suspension was centrifuged (10,000× *g*, 10 min, 4 °C) and sonicated (Hielscher Co. Teltow, Germany) with short pulses (30 pulses of 30 s with a 1-min pause between them, amplitude 80%). The debris were removed by centrifugation (10,000× *g*, 10 min, 4 °C) and the supernatant containing soluble protein was subjected to affinity chromatography using a Ni-NTA agarose column (Qiagen Co., Hilden, Germany). The Ni-NTA agarose column was washed and equilibrated by washing buffer (50 mM sodium phosphate buffer, 300 mM NaCl, and 20 mM imidazole at a pH of 8.0). The sample was loaded onto the column and rinsed by washing buffer as mentioned above to remove the impurities. His-tagged protein was eluted with elution buffer (250 mM imidazole in PBS 1X). The purified protein was then dialyzed at 4 °C with PBS 1X (pH 7.4) and analyzed on SDS-PAGE.

### 2.10. Western Blotting

Recombinant protein was run on 15% SDS-PAGE in reducing condition. The protein was then transferred to the nitrocellulose membrane by a semi-dry blotting system (Bio-Rad Co. Hercules, CA, USA). The nitrocellulose membrane was blocked using skim milk (4%) at room temperature (RT) for 1 h and incubated with mice monoclonal anti-poly histidine antibody at titer of 1:1000 at RT for 1 h, followed by washing three times with phosphate-buffered saline with 0.5% Tween (PBST). NC membranes were incubated with goat peroxidase-conjugated anti-mouse IgG antibody (Sigma-Aldrich, 1:2000) as a secondary antibody at RT for 1 h, and after washing were developed by 3,3′-Diaminobenzidine (DAB).

### 2.11. Evaluation of the Phospholipase Activity of Purified Leptulipin

Phospholipase activity of leptulipin was determined using a colorimetric method as previously published [40]. Briefly, serial concentrations of the protein were prepared in a microplate including 50–0.39 µg/mL. Then, the substrate solution was added to each well and incubated at 37 °C for 15 min. PBS 1x was used as negative control and the crude venom from *H. lepturus* scorpion was used as positive control. Optical density was read at 550 nm in a microplate spectrophotometer (EPOCH, BioTeK Co., Winooski, VT, USA).

### 2.12. Evaluation of Anticancer Activity of Leptulipin

#### 2.12.1. MTT Assay

The cells including HEK-293 (human embryonic kidney), HT-29, and MDA-MB-231 were grown in DMEM supplemented with 10% heat inactivated fetal bovine serum, 10 μL/mL penicillin–streptomycin solution. HEK-293 cell line was used to evaluate the toxicity of leptulipin on normal cells. The cytotoxicity of leptulipin was assessed as aforementioned above. 

#### 2.12.2. Lactate Dehydrogenase Release Assay

The cancer cell lines were seeded in 96-well plates (3 × 10^4^ cells/well) and incubated with leptulipin at concentrations of 50, 25, 12.5, and 6.25 µg/mL at 37 °C for 24 h. After incubation, lactate dehydrogenase (LDH) activity was measured in the cell lysate and supernatants using in vitro toxicology assay kit (Pars Azmoon Co., Tehran, Iran) in accordance with the manufacturer’s instructions. The absorbance was determined at 340 nm using a microplate spectrophotometer (Epoch, BioTek Co. Winooski, VT, USA).

#### 2.12.3. Analysis of Cell Morphology

The cells were incubated with and without leptulipin for 24 h. Then, the cells were washed with PBS and fixed with 4% formaldehyde for 15 min, followed by washing two times with PBS and staining with Hoechst 33258 (10 μg/mL) for 20 min in dark at RT. The cells were then visualized under a fluorescence microscope (Zeiss Axioplan, Malente, Germany).

### 2.13. Action Mechanism of Leptulipin

#### 2.13.1. DNA Fragmentation Assay

The cells were treated with leptulipin for 24 h and centrifuged at 10,000 rpm for 10 min. The cells were washed twice with PBS, resuspended in 0.4 mL lysis buffer (100 mM Tris HCl, pH 8.0, 2 mM EDTA, 0.8% (*w/v*) SDS, proteinase K 500 mg/mL), and incubated at 37 °C overnight. Phenol/chloroform/isoamyl alcohol (25:24:1, *v/v/v*) was added to the extracted DNA and the precipitation step was performed by 1/10 volume of 3 M ammonium acetate and 2 volumes of absolute ethanol, followed by centrifugation (10,000 rpm, 15 min, 4 °C). The pellet was dried at RT for 30 min, resuspended in TE buffer (Tris–EDTA), and incubated with RNase A (100 mg/mL) for 30 min. DNA fragmentation was analyzed by electrophoresis on a 1.5% agarose gel at 60 V for 4 h and visualized under UV light.

#### 2.13.2. Expression of *Casp9*, *Bax*, and *Bcl-2* Genes

The cells were treated with leptulipin for 24 h, harvested, and used for total RNA extraction. Reverse transcription PCR (RT-PCR) was then performed to analyze the expression of apoptosis-related genes (*Bax*, *Bcl-2*, *Casp9*). Briefly, the cells were subjected to RNA extraction using an RNA extraction kit according to the manufacturer instruction (RNX-Plus, Cinnagen Co., Tehran, Iran). cDNA was synthesized using the QuantiTect Reverse Transcription Kit (Qiagen Co., Hilden, Germany) and RT reactions were performed using a real-time thermal cycler (Rotor-Gene™ 6000, Corbett Life Science, Sydney, Australia). The primers for *Casp9*, *Bax*, and *Bcl-2* are depicted in Table 1. The house keeping gene, glyceraldehyde-3-phosphate dehydrogenase (*GAPDH*), was used as an internal reference gene to normalize the expression of the apoptotic genes (Table 1).

#### 2.13.3. Cell Cycle Arrest by Flow cytometry

The cells (1 × 10^6^ in a 6-well plate) were treated with leptulipin for 24 h, trypsinized, washed with cold PBS, and centrifuged. The cell pellet was resuspended in 500 μL PBS and incubated with 5 μL RNase A (20 μg/mL final concentration) for 20 min. The cells were incubated with propidium iodide (50 μg/mL final concentration) for an additional 30 min in the dark. The cells were then washed with PBS and immediately analyzed using flow cytometry in order to determine cell cycle phase. Untreated cells were used as control.

### 2.14. Statistical Analysis

Statistical analyses were performed by GraphPad Prism 9 software (San Diego, CA, USA). All experiments were done in triplicate and data are expressed as mean ± standard deviation (SD). The differences between the treated and control groups were analyzed by one-way ANOVA, and *p*-values ≤ 0.05 were considered relevant.

## 3. Results

### 3.1. Isolation of Anticancer Compound and Cytotoxicity Studies

**Figure 1:**
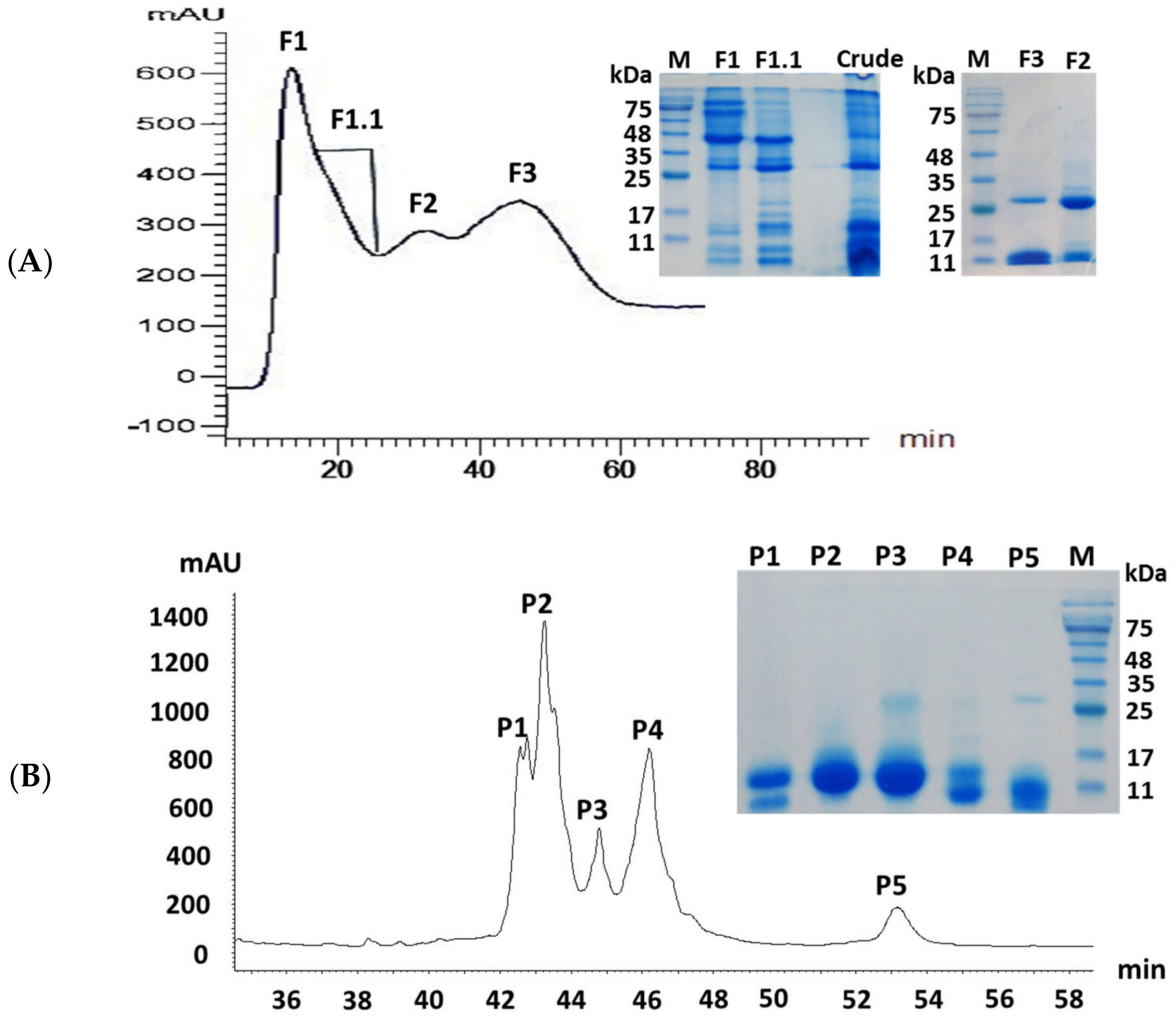
Fractionation of *H. lepturus* venom. (**A**) Gel filtration of crude venom on the Sephadex G50 HR column showed four fractions. (**B**) Further purification of the anticancer fraction, F3, by RP-HPLC provided five fractions. All fractions were analyzed by 12% SDS-PAGE.

**Figure 2:**
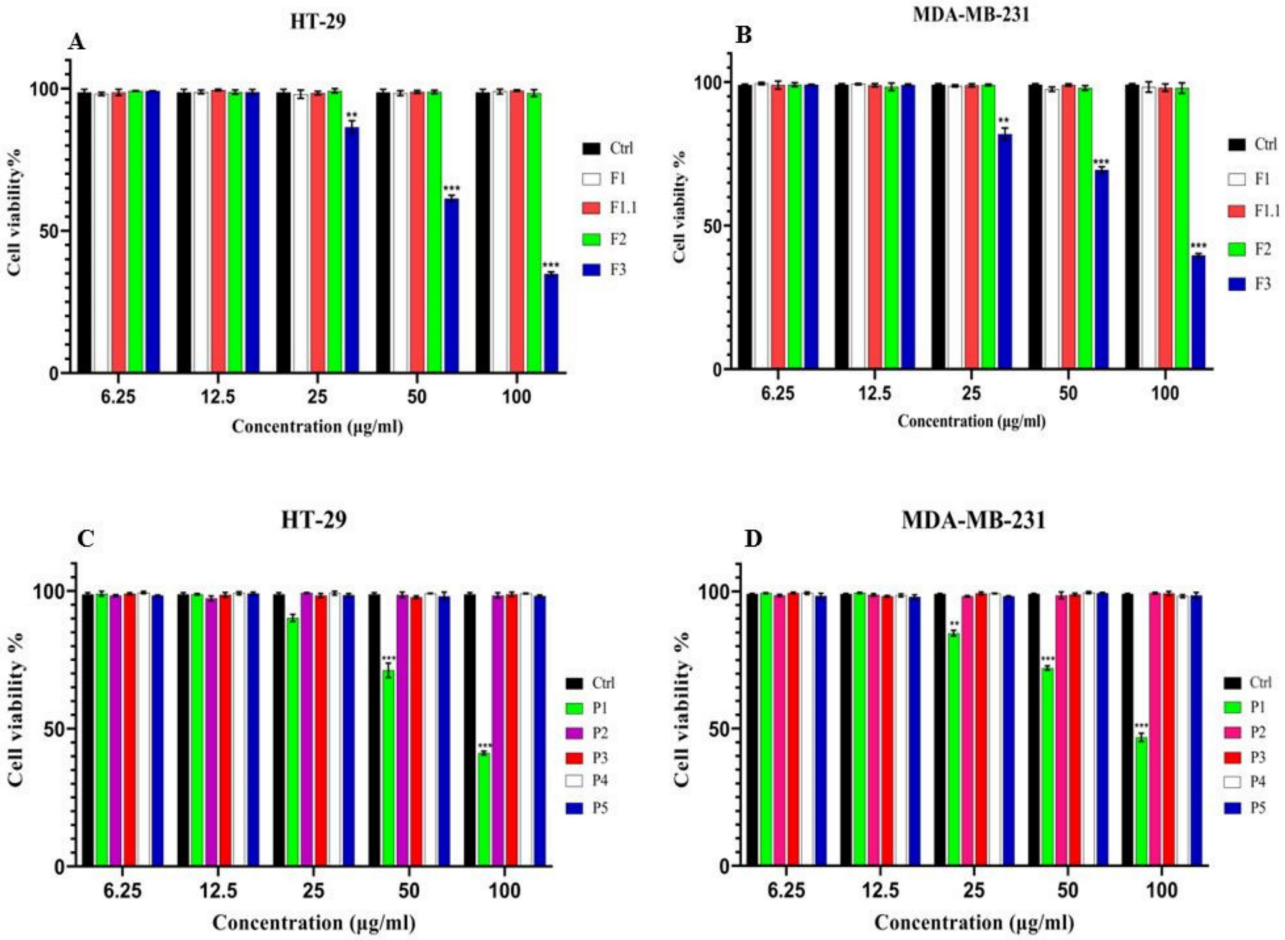
Cell viability of HT-29 and MDA-MB-231 cell lines. Fraction F3 obtained by gel-filtration chromatography showed the maximum anticancer activity (**A**,**B**). Amongst RP-HPLC- derived fractions, Fraction P1 showed the highest cytotoxicity against both cell lines (**C**,**D**); N = 3, *p* < 0.01 (**) and *p* < 0.001 (***).

To isolate an anticancer compound, bio-guided fractionation including gel filtration and reversed- phase chromatography was performed. In the first step, gel-filtration on Sephadex G50 column was used and four fractions were obtained (Figure 1A). The fractions were concentrated and subsequently applied to cytotoxicity assay against HT-29 and MDA-MB-231 cell lines. Only fraction F3 showed cytotoxicity on both cell lines at a concentration of 100 µg/mL. The percentage of cell viability of HT-29 and MDA-MB-231 cells was decreased significantly to 34.8 and 39.6%, respectively, in comparison to control (Figure 2A,B). Further separation of fraction F3 by RP-HPLC using a C8 column mainly revealed 5 peaks (Figure 1B). To evaluate cytotoxicity, MTT assay was done for the HPLC-derived fractions. As a result, only fraction P1 showed cytotoxicity against both cell lines. One hundred µg/mL of fraction P1 reduced the viability of HT-29 and MDA-MB-231 cells to 41.2 and 46.8%, respectively, in comparison to control (Figure 2C,D).

### 3.2. Protein Identification

**Figure 3:**
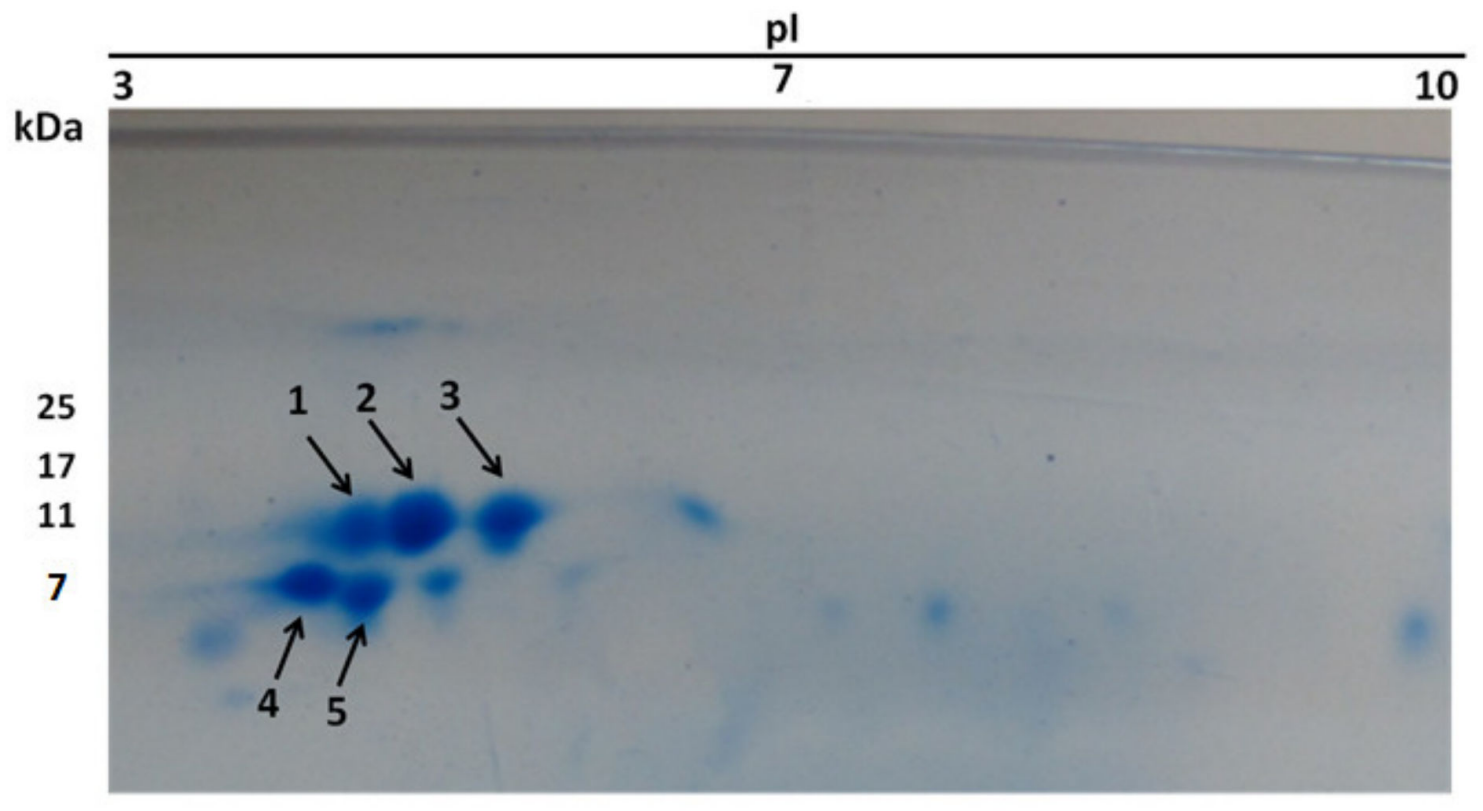
Two-dimensional gel electrophoresis of the HPLC-derived-fraction, F3. Five identical spots were selected and partial sequencing was performed. The numbers 1–5 refer to the identical protein spots.

In the next step, to identify the anticancer protein in the purified fraction, it was subjected to 2D electrophoresis (Figure 3). Five identical spots were seen (Figure 3).

The resultant spots were collected from the gel and subjected to in-gel digestion followed by MALDI-TOF-TOF. Peptide Fragments 1 and 2 corresponding to spot 1 were completely matched to venom toxin from *Hemiscorpius lepturus* (accession number: API81337). BlastP analysis of the full length sequence of venom toxin showed that it is a PLA2 molecule (Table 2).

Peptide fragments corresponding to spots 2, 3, and 5 were partially matched to other PLA2s from scorpions and other taxa. New PLA2 sequences from cDNA library of *Hemiscorpius lepturus* (accn: SRX1584448) were manually extracted. BlastP for peptide fragments derived from spots 2, 3, and 5 against each of extracted sequence showed that they matched completely with those PLA2 sequences in cDNA library. Accordingly, spots 2, 3, and 5 were determined as PLA2 (Table 2). The peptide fragment relating to spot 4 did not match to any proteins in scorpions (taxid: 6855).

**Scheme 2:**
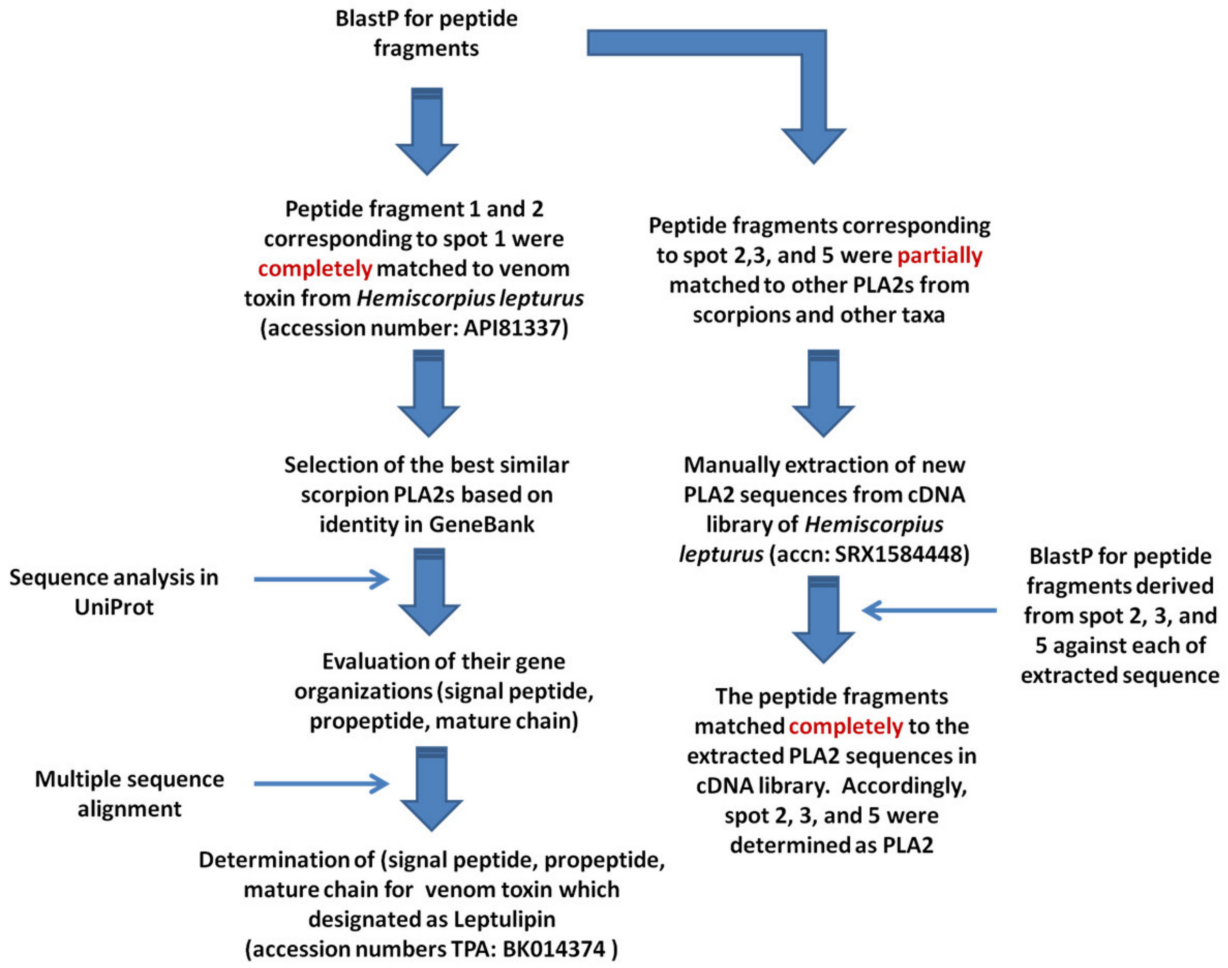
The diagram of bioinformatics analyses for identification of the peptide fragments, selection of a PLA2 candidate, as well as determination of the mature chain for cloning.

The diagram of bioinformatics analyses for identification of the peptide fragments is depicted in Scheme 2.

### 3.3. Bioinformatics Analysis

#### 3.3.1. Similarity Analyses

In reference to BlastP analysis, all the peptide fragments corresponding to Proteins 1, 2, 3, and 5 hit to PLA2 sequences, which had already been documented in the cDNA library of *H. lepturus* (accn: SRX1584448, https://www.ncbi.nlm.nih.gov/sra/SRX1584448 accessed on 12 August 2021). Those abovementioned sequences are unique and have not been deposited to GenBank yet except for Protein 1 which has been already deposited as venom toxin (protein accession number: API81337).

All the full length sequences corresponding to spots 1, 2, 3, and 5 were analyzed by multiple alignments against the scorpion PLA2 family to deduce their gene organization (Signal peptide, propeptide, and mature chain). The result was satisfactory for only spot 1. Accordingly, the venom toxin was selected for further characterization and hereafter designated as leptulipin (accession number TPA: BK059885). The sequences of peptide fragments corresponding to the protein hits are listed in Table 2. The diagram of bioinformatics analysis for identification of the peptide fragments, selection of a PLA2 candidate, as well as determination of the mature chain is depicted in Scheme 2.

#### 3.3.2. Determination of the Mature Chain

**Figure 4:**
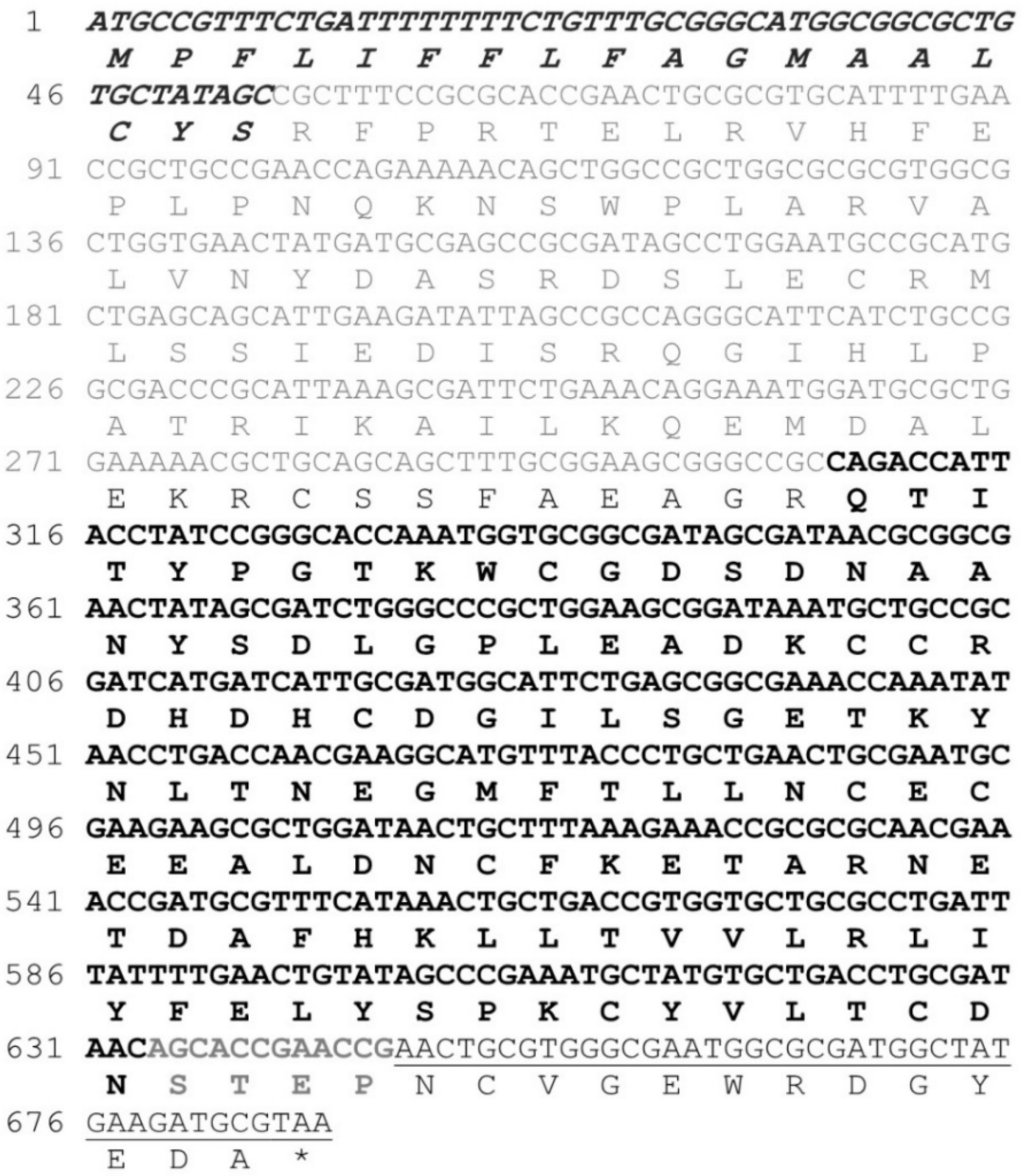
The amino acid and ORF sequence of leptulipin. Signal peptide: italic; propeptide: gray; mature chain: black; propeptide: gray, small subunit: underlined sequence.

According to the similarity between the full sequence of leptulipin and phospholipin, hemilipin, *Scorpio maurus* PLA2, and phaiodactylipin (Table 3), the signal peptide, propeptide, small subunit, and the mature chain were deduced (Figure 4).

#### 3.3.3. Similarity Analyses

**Figure 5:**
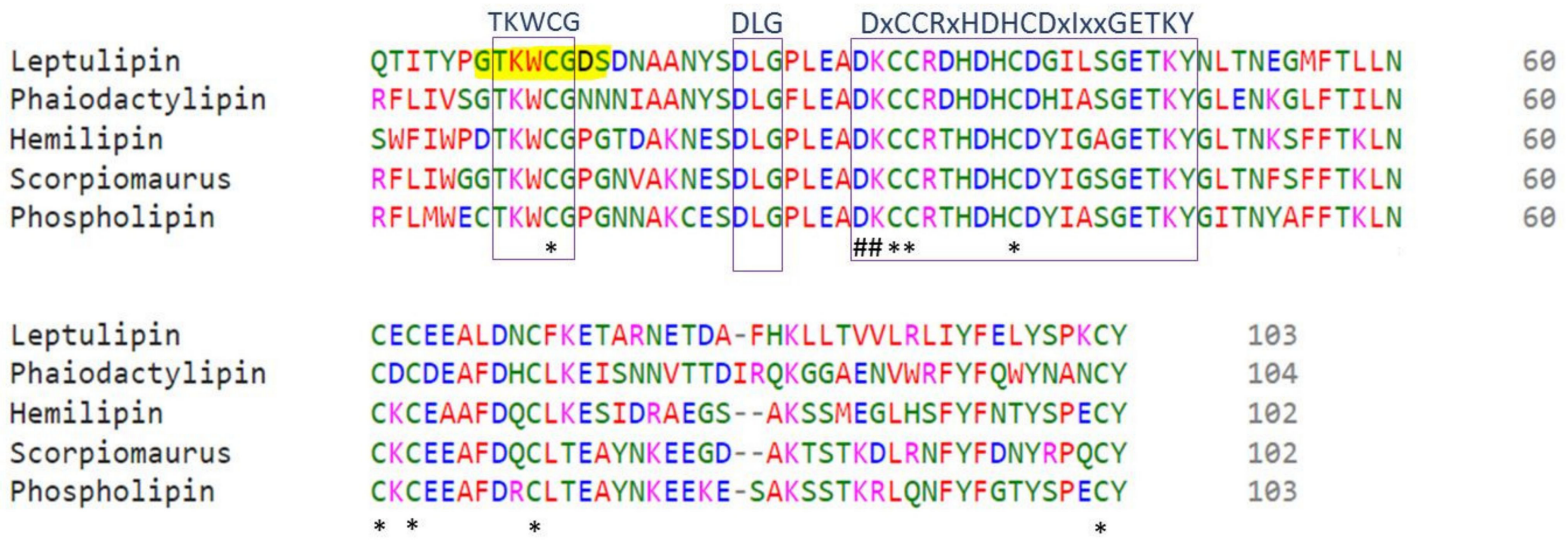
Multiple sequence alignment of leptulipin with other scorpion PLA2 sequences (phospholipin, phaiodactylipin, *Scorpio maurus* PLA2, and hemilipin) by Clustal Omega. Boxes show the evolutionary signature in the selected similar sequences as TKWCG, DLG, and DxCCRxHDHCDxIxxGETKY. Conserved cysteine and the main catalytic residues are represented by * and #, respectively. The yellow box shows the calcium-binding loop.

Furthermore, to characterize the conserved regions, active site, and evolutionary signature of leptulipin, its similarity with other scorpion PLA2 was determined (Figure 5) using multiple alignment by Clustal Omega [41].

#### 3.3.4. Prediction of 3D and Structural Alignment

**Figure 6:**
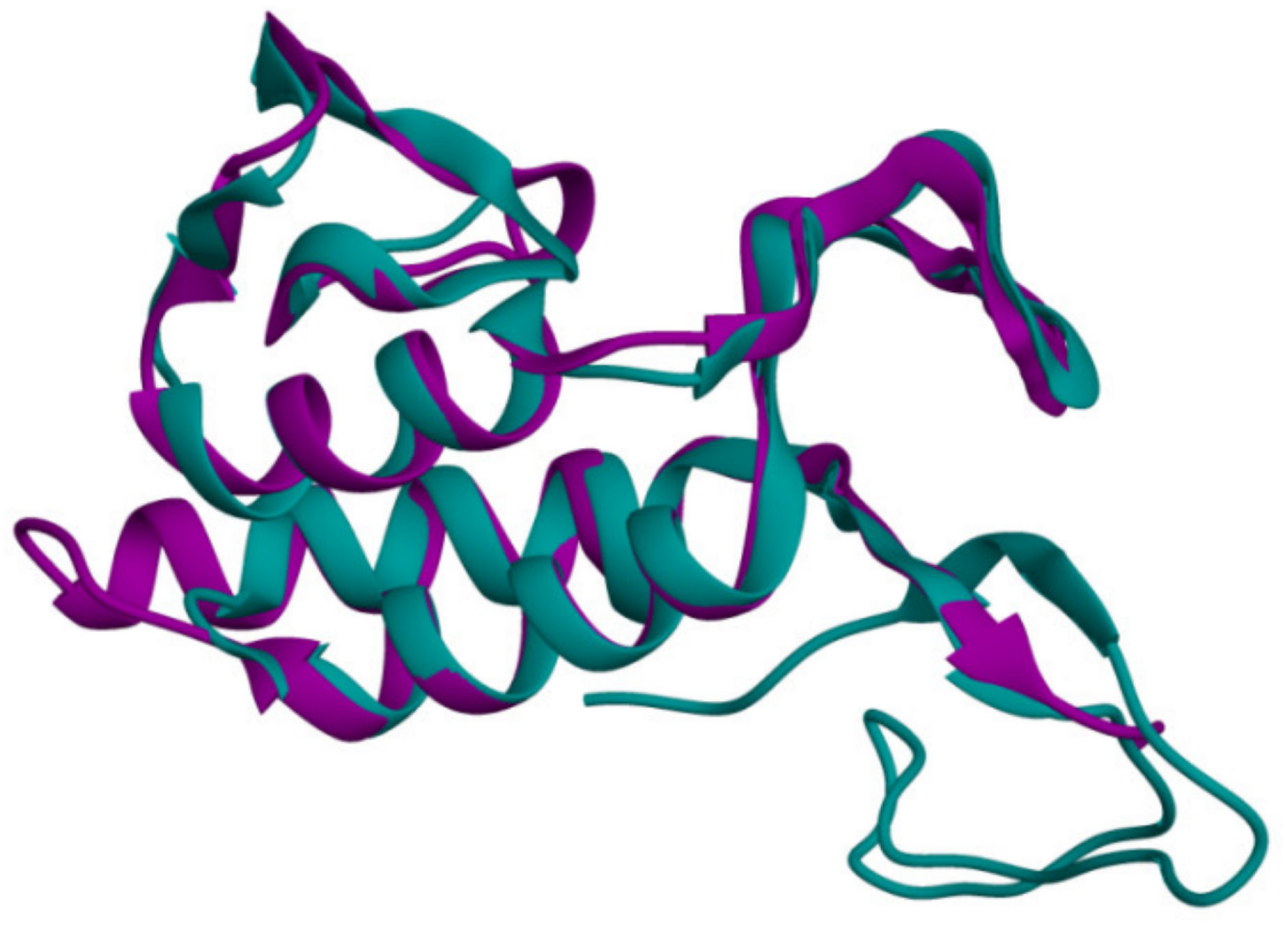
Superimposition of the 3D structure of leptulipin (magenta) with the honey bee PLA2 (cyan), showing that both toxins significantly match.

The predicted structure of leptulipin contains eight coils, four sheets, and three helices (Figure 6). It includes 28.44% α-helices, 10.09% β-sheets, and 61.47% random coils. The structural alignment of leptulipin with honey bee PLA2 (PDB 1POC) using the I-Tasser server showed a significant similarity (C-score = 0.62, RMSD = 2.9 ± 2.1 Å). The structural alignment was visualized by the UCSF Chimera software package.

### 3.4. Molecular Cloning, Purification, and Western Blotting of Leptulipin

**Figure 7:**
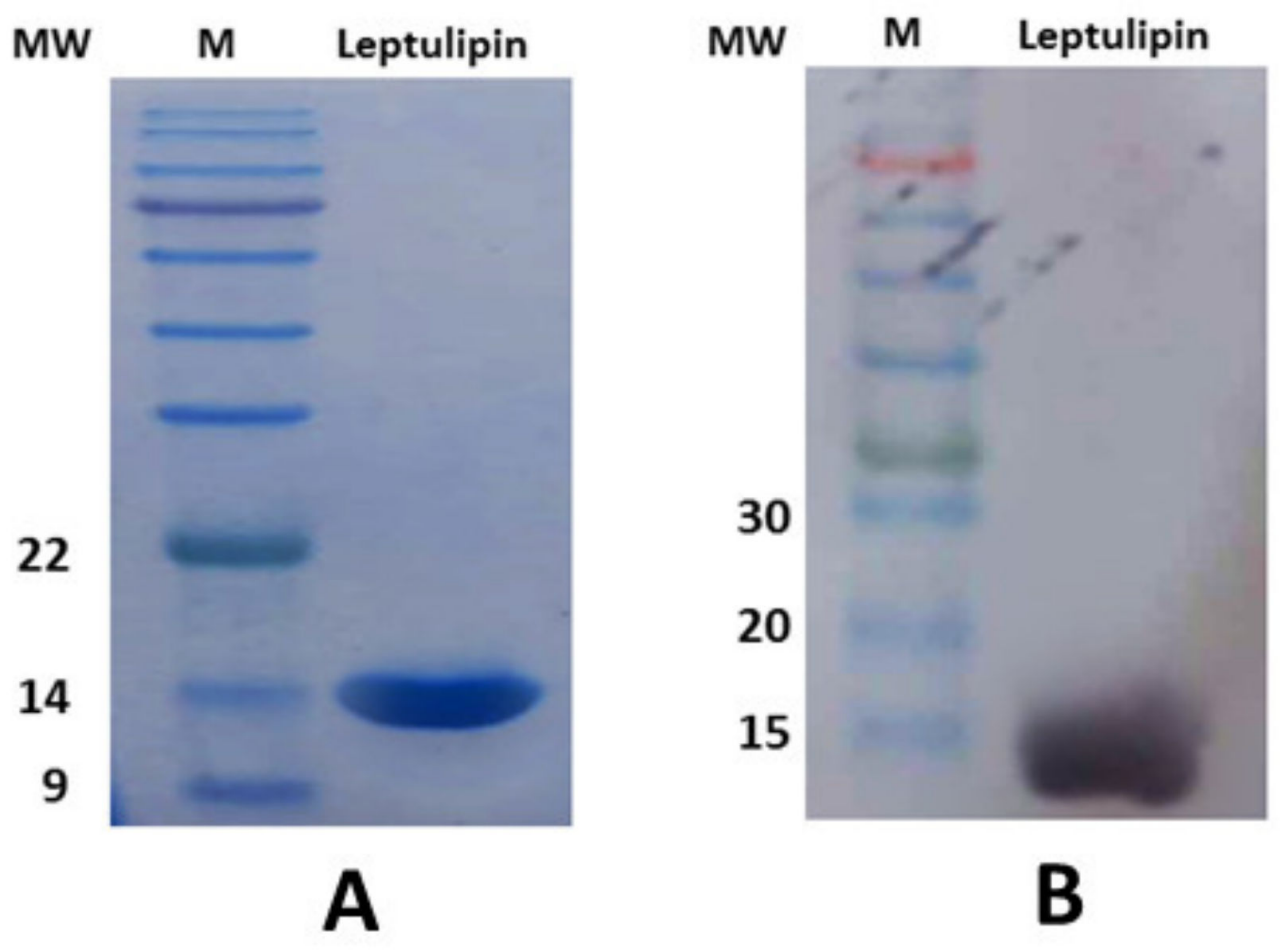
(**A**) SDS-PAGE and (**B**) western blotting of the expressed and purified leptulipin. Purified leptulipin was detected at the molecular weight of ca. 14 kDa.

The full-length sequence of the large subunit of leptulipin was inserted to pET-26b and the expression of leptulipin showed a single purified protein at the molecular weight of ~14 kDa in SDS-PAGE (Figure 7A). Purified recombinant protein analysis by western blot showed that the 14-kDa band is in agreement with the expected molecular weight for the expressed recombinant protein of leptulipin (Figure 7B).

### 3.5. Enzymatic Activity Assay for Leptulipin

**Figure 8:**
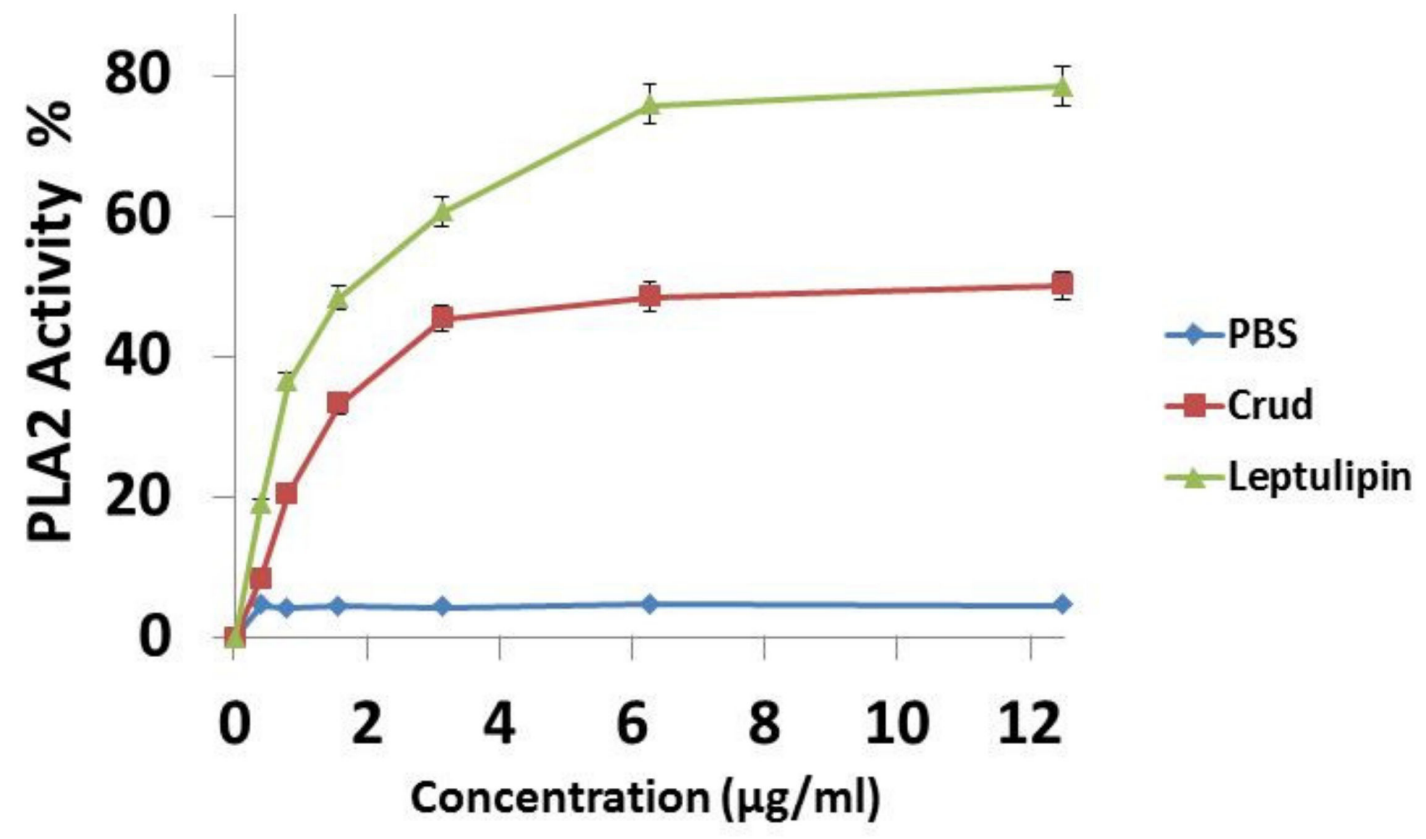
Evaluation of PLA2 activity. Various concentrations of leptulipin and crude venom were incubated with reaction mixture containing 3.5 mM lecithin. The PLA2 activity percentage was calculated from the phenol red absorbance variations due to the fatty acid release. PBS was used as a negative control. Leptulipin reached its maximum activity at ca. 6.25 µg/mL (N = 3).

As shown in Figure 8, leptulipin was able to hydrolyze lecithin. Leptulipin showed high phospholipase activity, higher than the crude venom at the same concentrations. Leptulipin reached to its maximum activity at ca. 6.25 µg/mL.

### 3.6. Inhibition of Cell Proliferation

**Figure 9:**
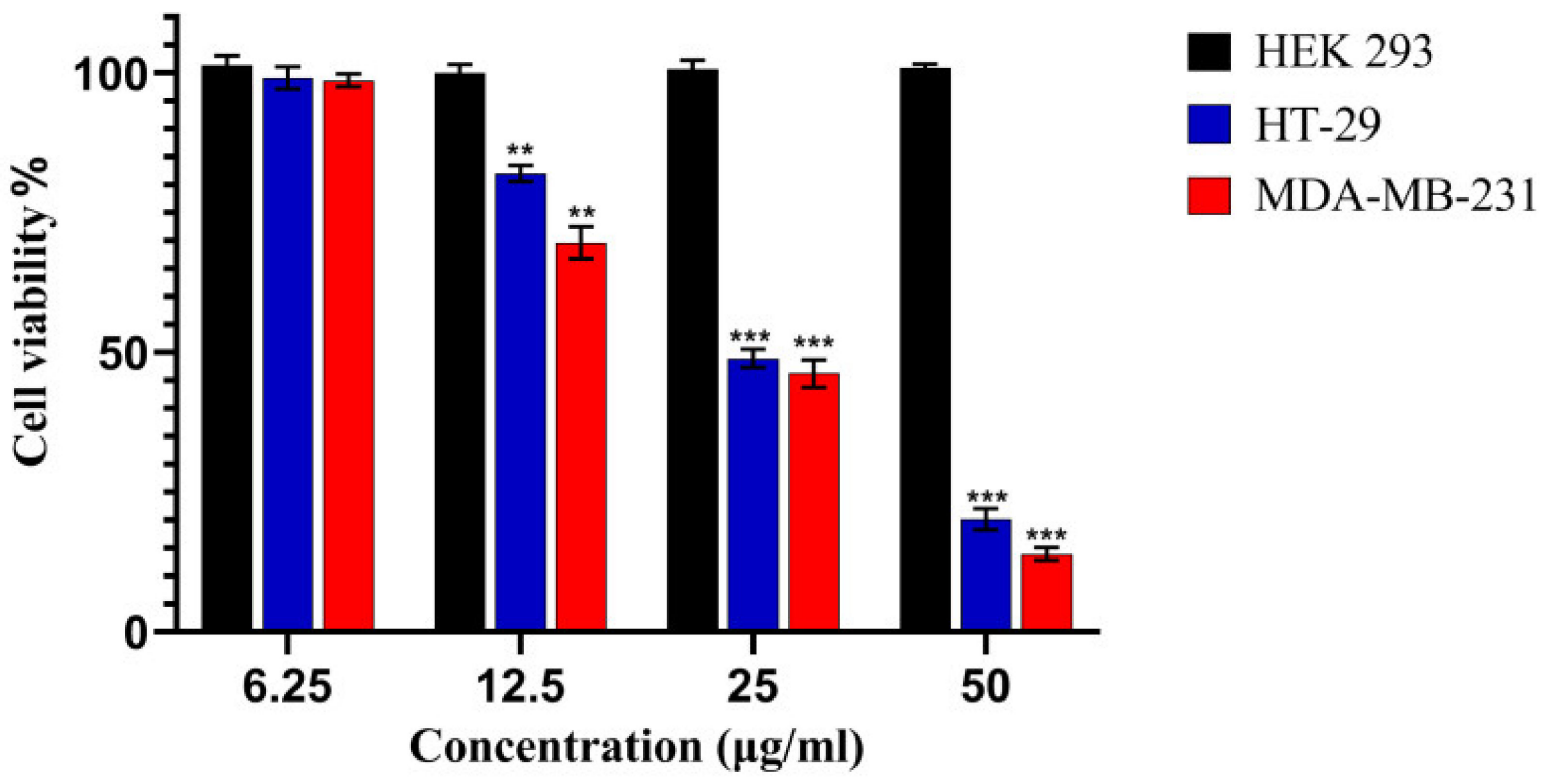
Assessment of the inhibition of cell proliferation by MTT assay. The cell lines were treated by serially diluted concentrations of leptulipin for 24 h. The maximum inhibition of cell proliferation was seen at 50 µg/mL. N = 3, *p* < 0.01 (**) and *p* < 0.001 (***).

The effect of leptulipin on cell viability was evaluated using MTT and LDH release assays (Figure 9). Leptulipin significantly inhibited cell growth in a dose-dependent manner. Leptulipin at 50 µg/mL inhibited the proliferation of HT-29 and MDA-MB-231, at 20.13 ± 1.9% and 13.91 ± 1.17%, respectively. IC_50_ value was around 40 and 30 µg/mL for HT-29 and MBA-231 cell lines, respectively, over 24 h. No toxicity was seen on HEK 293 as a normal cell at all examined concentrations.

### 3.7. LDH Release Assay

**Figure 10:**
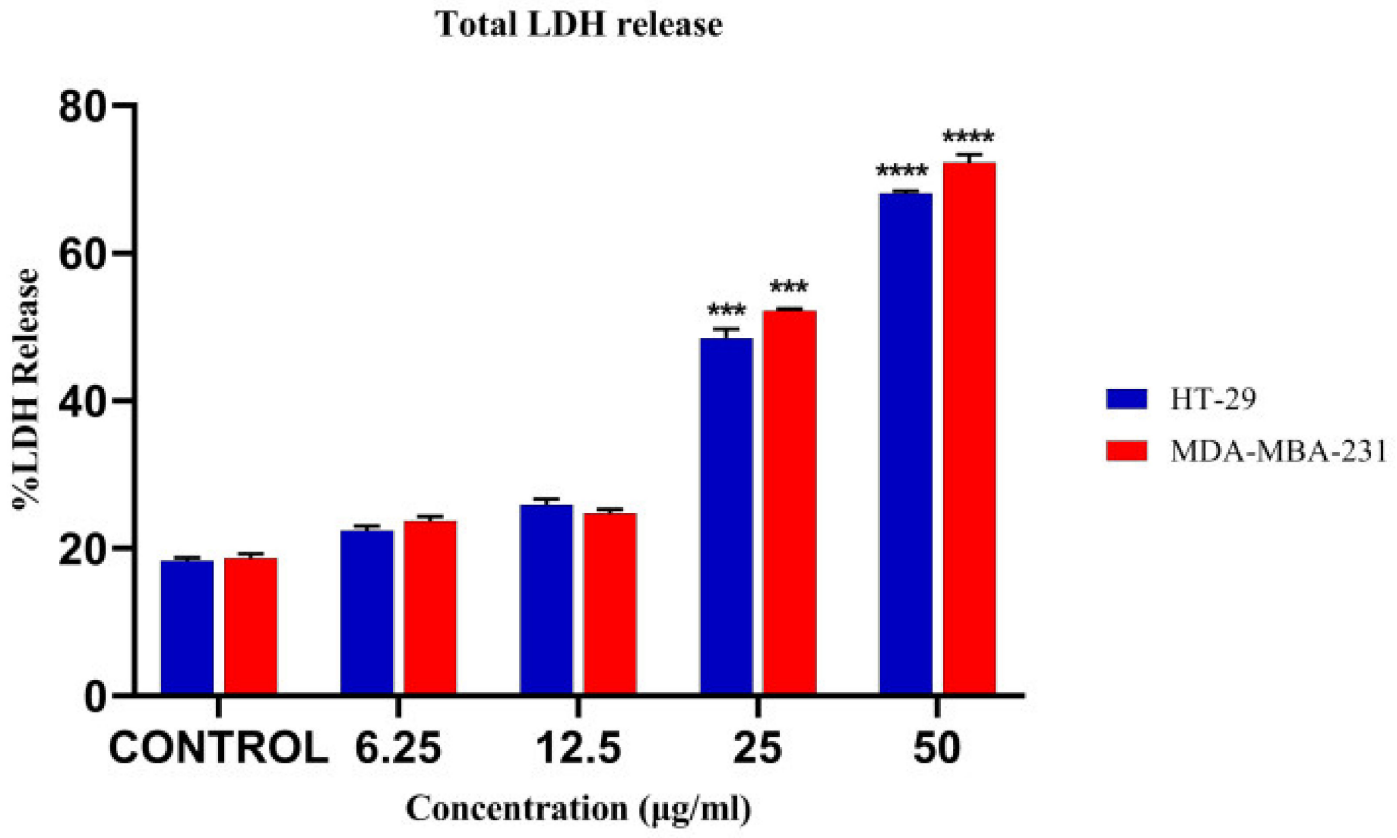
Effects of leptulipin on LDH release from the cancer cells. Maximum release was seen at 50 µg/mL. N = 3, *p* < 0.001 (***) and *p* < 0.0001 (****).

The significant LDH release was detected at the concentration of 50 µg/mL from the cell lines in comparison to the negative control (Figure 10).

### 3.8. Effect of Leptulipin on Cell Morphology

**Figure 11:**
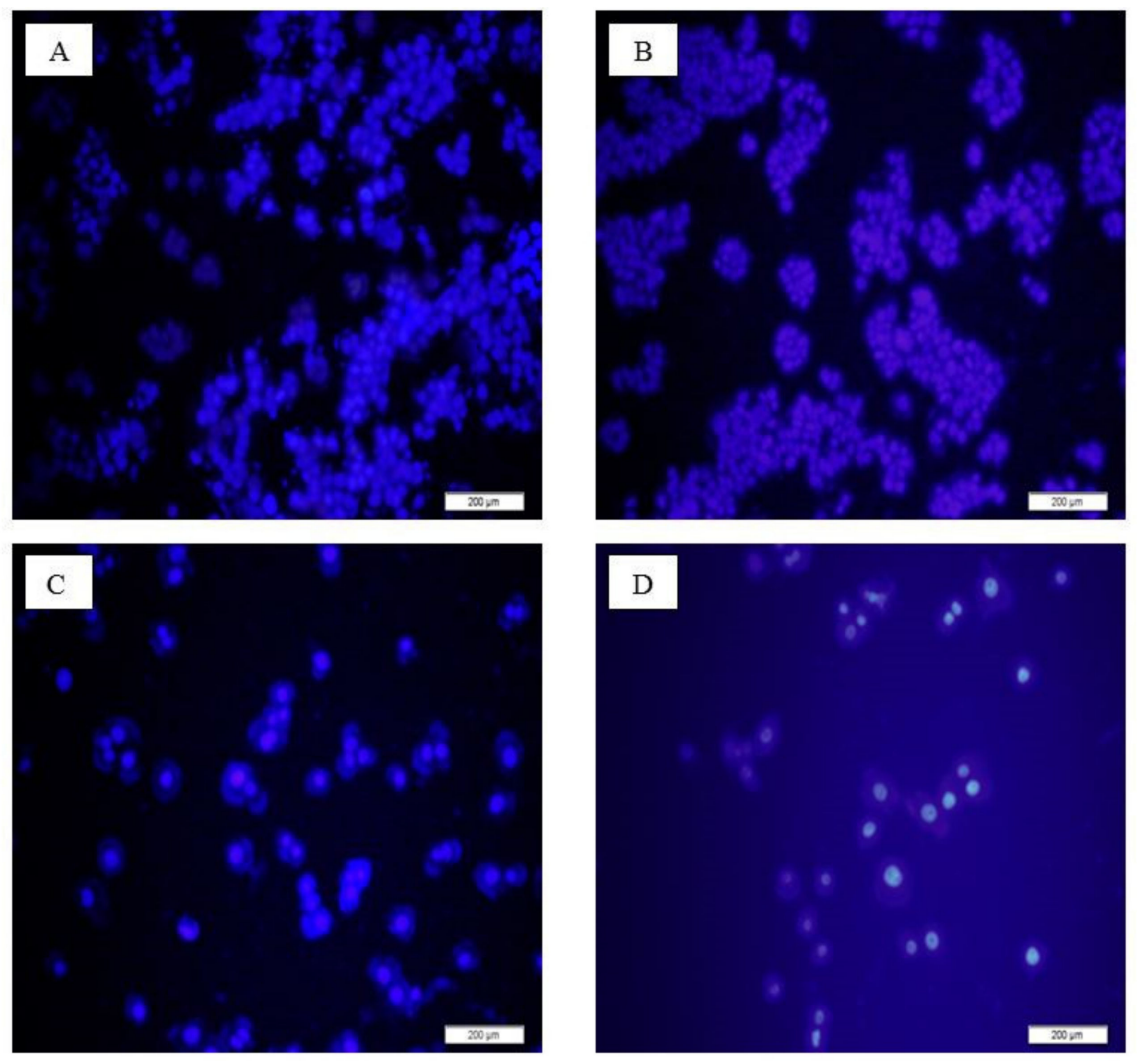
Morphological alterations of cancer cells. Hoechst 33258 staining showed morphological alterations such as shrinkage, loss of cell integrity, and chromatin condensation ((**C**,**D**): HT-29 and MDA-MB-231) in comparison to untreated cells ((**A**,**B**): HT-29 and MDA-MB-231).

The treated cells showed morphological alterations such as shrinkage, loss of cell integrity, and chromatin condensation in comparison to untreated cells (Figure 11).

### 3.9. Analysis of Cell Cycle Arrest

**Figure 12:**
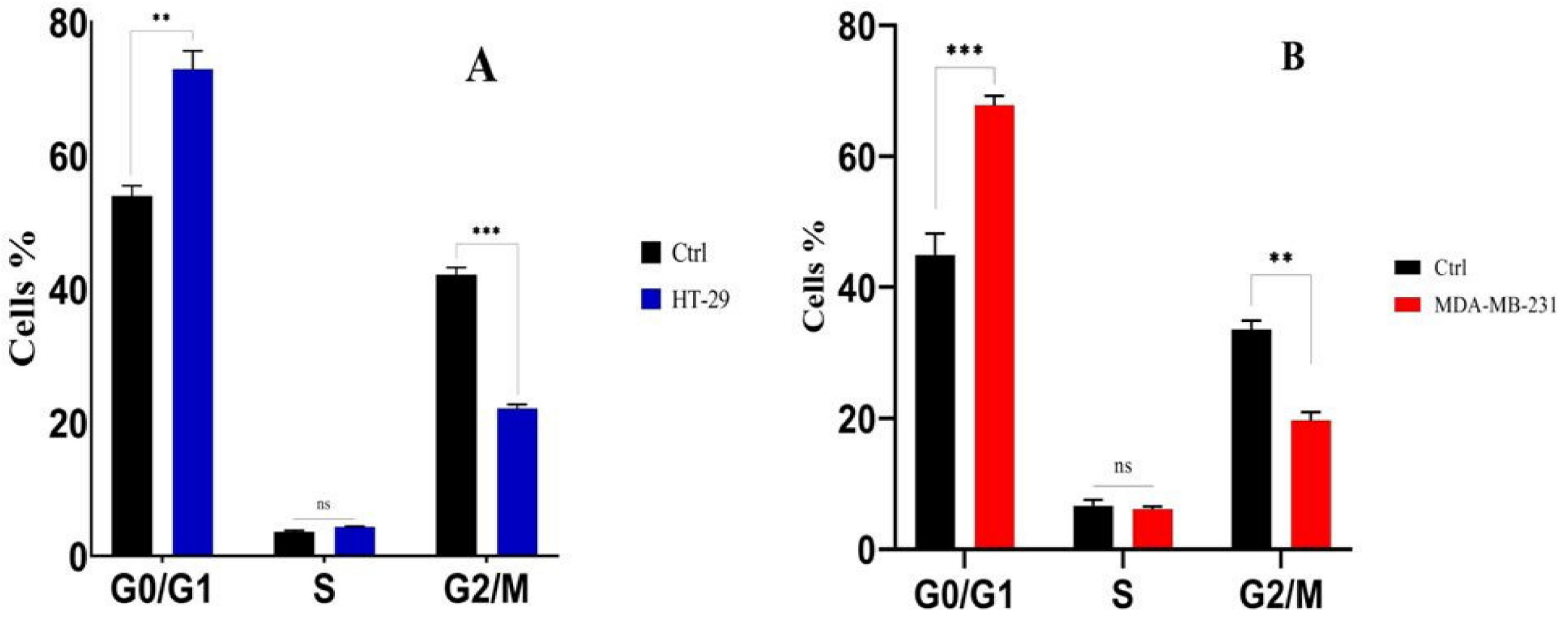
Evaluation of cell cycle phases in HT-29 and MDA-MB-231 cells after 24 h treatment. Leptulipin increased the number of cells in the G0/G1 phase and decreased the number of cells in the G2/M phase. (**A**) HT-29 and control. (**B**) MDA-MB-231 and control. N = 3, *p* < 0.01 (**), and *p* < 0.001 (***).

The treatment of HT-29 cells and MDA-MBA123 with leptulipin resulted in a 67.8 and 72.9% increase in the G0/G1 phase and a 22.1 and 19.6% decrease in G2/M phase, respectively, whereas the percentage of treated cells in the S phase was similar to the control cells (Figure 12).

### 3.10. DNA Fragmentation Analysis

**Figure 13:**
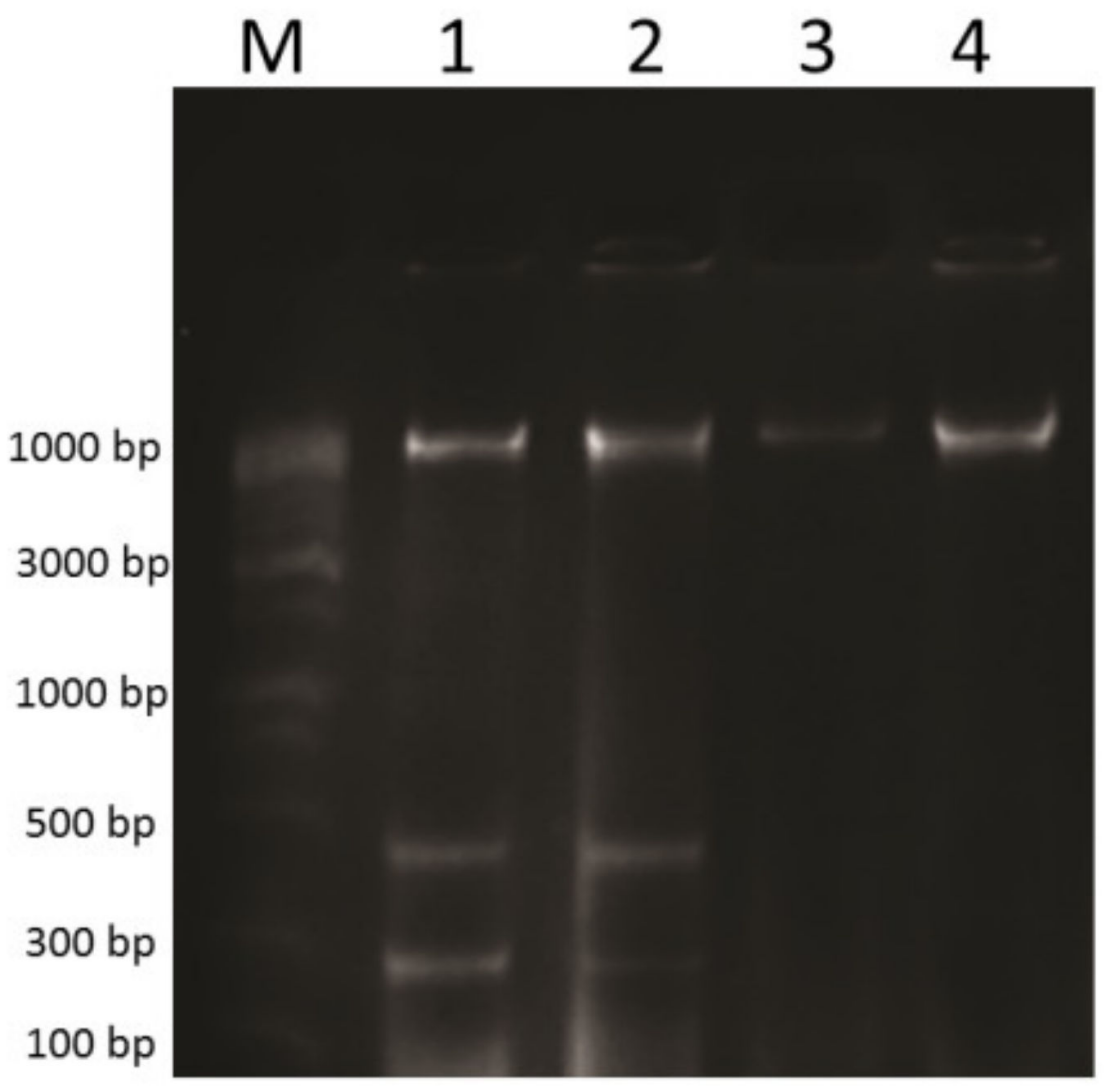
DNA fragmentation analysis of HT-29 and MDA-MBA123 cells after 24 h treatment with leptulipin. M—DNA marker. 1—HT-29 cells treated with 25 μg/mL of leptulipin. 2—MDA-MBA123 treated cells with 25 μg/mL of leptulipin. 3 and 4—untreated HT-29 and MDA-MBA123 cells.

The results indicated that leptulipin induced DNA fragmentation in the treated cancer cell lines. DNA fragmentation was observed in 200–400 bp regions as compared to the control cells (Figure 13).

### 3.11. Expression of Bax, Bcl-2 and Casp9

**Figure 14:**
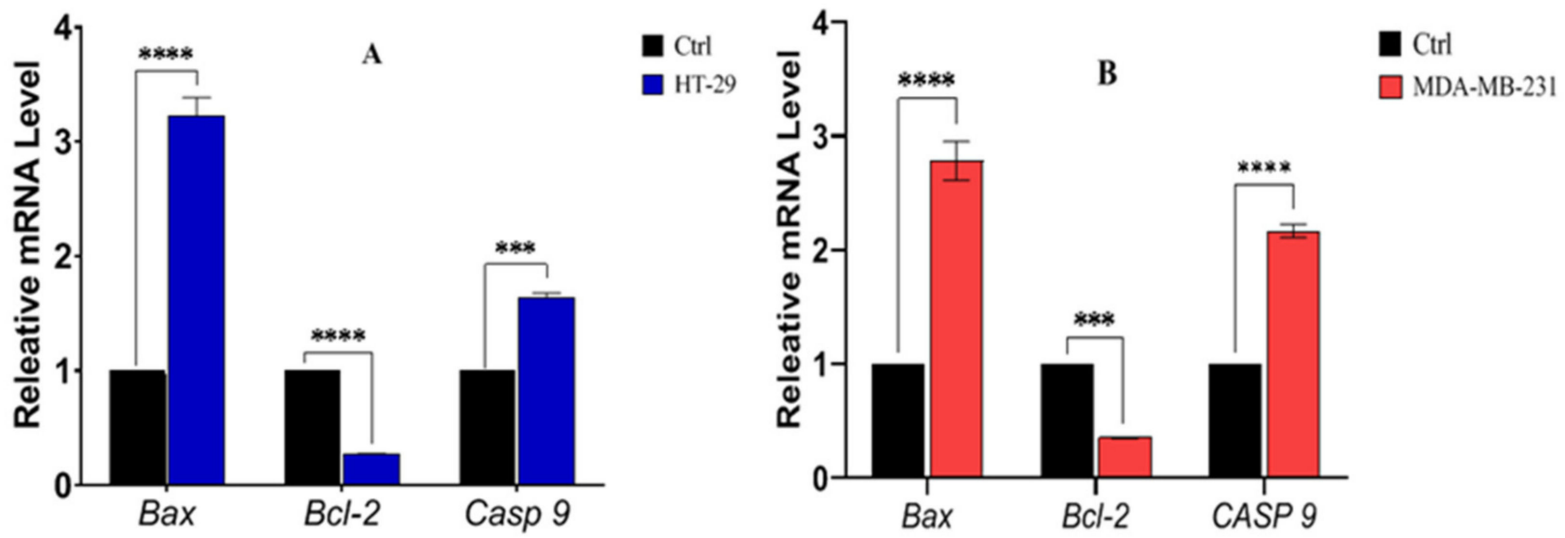
The expression of *Bax*, *Bcl-2*, and *Casp9* genes. (**A**) HT-29 cells and (**B**) MDA-MB-231 cells. N = 3, *** *p* < 0.001, **** *p* < 0.0001.

In two cell lines, the expression of *Bax* and *Casp9* mRNA was upregulated significantly after 24 h of treatment compared to untreated controls. On the other hand, *Bcl-2* mRNA expression was significantly down-regulated in the treated group in comparison to control cells (Figure 14).

## 4. Discussion

The pharmaceutical industry strives to discover new drugs for cancer therapy because it is one of the leading causes of morbidity and mortality worldwide [42,43]. Scorpion venoms are composed of a wide variety of enzymatic and non-enzymatic molecules involved in defense mechanisms [44]. They have a range of bioactive compounds with anticancer potential that target mainly ion channels and membrane receptors with high selectivity and affinity [45,46,47]. Proteomics, transcriptomics, as well as bioinformatic tools have been used to find new putative leads based on naturally produced toxins [48,49].

The purpose of our study was the identification of the most effective anticancer compound from the venom of *H. lepturus* scorpion, an extremely toxic species found in the south and southwest of Iran [26]. The first step of the discovery process was the isolation of the active compound from the venom using an activity-guided purification method. After gel-filtration, a dominant peak was obtained (Fraction F3). This fraction was cytotoxic to cancer cell lines. Then, the cytotoxic Fraction F3 was separated into three major fractions through reversed-phase chromatography. HPLC Fraction P1 was shown to be the only one to be cytotoxic.

In addition, to analyzing the protein profile of this fraction, 2D-PAGE was conducted. Based on LC-MS/MS analysis spot, a protein with a molecular weight of 14 kDa was identified. Homologous sequence search confirmed that this sequence belongs to the PLA2 family. As a result of proteomics and bioinformatics analyses, the main chain of leptulipin was cloned into pET-26b. It was expressed and purified. PLA2 activity was demonstrated in vitro by the PLA2 activity assay. Finally, we have studied the effects of leptulipin on inducing apoptosis on cancer cell lines.

Human colon and breast cancers are among the deadliest cancers [1]. Finding a new effective drug to treat colon and breast cancer is greatly needed. Scorpion venom has a powerful antitumor effect and their compounds are cytotoxic to several cancer cell lines [15,50,51,52]. In this study, we showed that leptulipin inhibits the cell proliferation of MDA-MB-231 and HT-29 cancer cells and that it increases LDH cytotoxicity. In other studies, similar observations have been reported, with cell proliferation reduced by up to 98% in some cancer cell lines with TsAP-S1 and TsAP-S2, two toxins isolated from the venom of *Tityus serrulatus* [53]. Treatment of HSC-4 and SW620 cells by BmKn2 (a protein isolated from *B. martensii* Karsch venom) induced 95% cytotoxicity for both [54]. Another study found that a bengalin inhibited the growth of U-937 and K-562 cell lines (48% and 50% respectively) [23]. Furthermore, Zhao et al. showed that rAGAP displayed anti-proliferative activity against SW-480 [24].

Apoptosis is the anticancer mechanism, which leads to a cascade of intracellular events and ultimately to cell death. Its pathways are both intrinsic and extrinsic. In the intrinsic pathway, an increase of the *Bax/Bcl-2* ratio is considered to promote the release of Cytochrome c from mitochondria. A high ratio of *Bax/Bcl-2* leads to greater activity of apoptosis [55,56]. The *Bax/Bcl-2* ratio was significantly increased in our study. Caspase cascades ultimately activate *Casp3*, which modulates distinct events, such as nuclear condensation and DNA fragmentation [55,56]. Our data indicate that the expression levels of *Bax* and *Casp9* mRNA were significantly increased after 24 h, while *Bcl-2* mRNA expression was significantly decreased in cells treated with leptulipin compared to the control.

Furthermore, using an agarose gel, we investigated whether *Casp3* causes the fragmentation of DNA in cells after treatment with leptulipin. DNA fragments found in the analysis confirmed apoptosis. The results indicated that leptulipin induces the intrinsic pathway of apoptosis although a comprehensive study should be performed to decipher the exact action mechanism of leptulipin. MjTX-I, a PLA2 from Bothrops moojeni snake venom, lowered the expression level of the anti-apoptotic gene BCL-2 and increased the expression level of the Bax gene [57]. Our result is in accordance with this study, proving the induction of apoptosis via the intrinsic pathway, while Pllans–II, an acidic PLA2 from Porthidium lansbergii snake venom, induces the extrinsic apoptosis pathway [58]. In this study, *Bax* and *Bcl-2* gene expression was decreased, whereas *Casp8* was increased [58].

BmKn-2 stimulated *Casp3* and *9* expression and down-regulated *Bcl-2* expression [54]. Overexpression of *Bax* was observed in cells treated with rAGAP as well [24]. Bengalin increased *Casp3* expression in cells. Apoptosis and cell cycle arrest are also closely linked [23].

Analysis of the data from cell cycle arrest indicated that leptulipin provokes cancer cells to be more active in G0/G1 phases, but suppresses cell activity in G2/M phases. Both rAGAP and bengalin induce cellular arrest in the G1 phase and sub-G1 phase, respectively [23,24].

Crotoxin, which is another PLA2 purified from the rattlesnake, was proposed as an anticancer drug and pushed through clinical trials [59]. Accordingly, we hope to develop leptulipin as a drug lead as well. Promising in vitro results showed that leptulipin can be a safe anticancer agent, although a comprehensive in vivo toxicity study should be performed.

We suggest a comprehensive analysis of apoptosis-related genes to decipher the exact action mechanism of leptulipin. The 3D structure of leptulipin should also be determined in order to study the structure–activity relationship.

## 5. Conclusions

In this study, we discovered an anticancer protein from *H. lepturus* scorpion venom. Leptulipin showed significant anticancer activity against breast and colon cancer cell lines through the inhibition of proliferation and cell cycle, as well as by inducing apoptosis and alteration in expression of anti- and pro-apoptosis genes. According to the promising anticancer activity of leptulipin, it could be considered as a new anticancer drug lead to be examined on animal models of breast and colon cancer.

## Figures and Tables

**Table 1:** Sequence of primers used to amplify *Casp 9*, Bax, *Bcl-2*, and *GAPDH* genes.

	HT-29	MDA-MB-231
*Casp 9* F	5′-CCAGAGATTCGCAAACCAGAGG-3′	5′-CTGTCTACGGCACAGATGGAT-3′
*Casp 9* R	5′-GGAGACCAAACGCTTAGAGACC-3′	5′-GGGACTCGTCTTCAGGGGAA-3′
*Bax* F	5′-AGGATGCGTCCACCAAGAAG-3′	5′-CCTGTGCACCAAGGTGCCGGAACT-3′
*Bax* R	5′-GGAAGAAGACCTCTCGGGG-3′	5′-CCACCCTGGTCTTGGATCCAGCCC-3′
*Bcl-2* F	5′-GACTTCGCAGAGATGTCCAGT-3′	5′-TTGTGGCCTTCTTTGAGTTCGGTG-3′
*Bcl-2* R	5′-CATCCCTGAAGAGTTCCTCCA-3′	5′-GGTGCCGGTTCAGGTACTCAGTCA-3′
*GAPDH* F	5′-GACCACAGTCCATGACATCACT	5′-CGGAGTCAACGGATTTGGTCGTAT-3′
*GAPDH* R	5′-TCACTACAGTACCTGACACCAG-3′	5′-AGCCTTCTCCATGGTGGTGAAGAC-3′

**Table 2:** The proteins identified from 2D electrophoresis spots.

Spot No.	Protein Name	Mass (kDa)	Peptide Sequence
1	Leptulipin	12.81	DHDHCDGILSGETK CEEALDNCFK
2	PLA2-2	12.09	KWCGAGSEA DHDHCDNIGAGETK
3	PLA2-3	12.32	CEDAFKQCLR THDHCDNIGSGETK
4	ND *	7.46	ND *
5	PLA2-4	7.38	DHDHCDNIIPGETK

* Not determined.

**Table 3:** Similarity analysis of the full sequence of leptulipin against other scorpion PLA2s.

Similar Protein	Identity	Accession Number	Organism Name	Reference
Phaiodactylipin	56.06%	Q6PXP0.2	*Anuroctonus phaiodactylus*	[36]
*Scorpio maurus* PLA2	50.34%	MF347455.1	*Scorpio maurus*	[31]
Phospholipin	48.30%	P0DKU2.1	*Pandinus imperator*	[37]
Hemilipin	46.35%	A0A1L4BJ46.1	*Hemiscorpius lepturus*	[30]

## Data Availability

The data presented in this study are available in the manuscript.

## Sample Availability

Samples of the compounds are not available from the authors.
